# The contemporary pulmonary artery catheter. Part 2: measurements, limitations, and clinical applications

**DOI:** 10.1007/s10877-021-00673-5

**Published:** 2021-03-01

**Authors:** I. T. Bootsma, E. C. Boerma, T. W. L. Scheeren, F. de Lange

**Affiliations:** 1 Department of Intensive Care, Medical Center Leeuwarden, Henri Dunantweg 2, P.O. Box 888, 8901 Leeuwarden, the Netherlands; 2 Department of Anesthesiology, University of Groningen, University Medical Center Groningen, Groningen, the Netherlands

**Keywords:** Hemodynamic monitoring, Pulmonary artery catheter, Thermodilution, Continuous cardiac output, Right ventricular ejection fraction, Right ventricular end-diastolic volume, Mixed venous oxygen saturation, Oxygen supply and -demand balance

## Abstract

Nowadays, the classical pulmonary artery catheter (PAC) has an almost 50-year-old history of its clinical use for hemodynamic monitoring. In recent years, the PAC evolved from a device that enabled intermittent cardiac output measurements in combination with static pressures to a monitoring tool that provides continuous data on cardiac output, oxygen supply and-demand balance, as well as right ventricular performance. In this review, which consists of two parts, we will introduce the difference between intermittent pulmonary artery thermodilution using bolus injections, and the contemporary PAC enabling continuous measurements by using a thermal filament which heats up the blood. In this second part, we will discuss in detail the measurements of the contemporary PAC, including continuous cardiac output measurement, right ventricular ejection fraction, end-diastolic volume index, and mixed venous oxygen saturation. Limitations of all of these measurements are highlighted as well. We conclude that thorough understanding of measurements obtained from the PAC is the first step in successful application of the PAC in daily clinical practice.

**Supplementary Information:**

The online version contains supplementary material available at 10.1007/s10877-021-00673-5.

## Introduction

Since the introduction of the original floating pulmonary artery catheter (PAC) by Swan and Ganz in 1970 the device has changed considerably. The classical PAC evolved from a catheter that enabled intermittent cardiac output (CO) measurements in combination with static pressures to a monitoring tool which provides continuous data on CO, the oxygen delivery and consumption balance, as well as right ventricular (RV) performance. Detailed understanding of the technology and its potential pitfalls are eminent in adequate interpretation of PAC-derived data. However, a large proportion of ICU physicians and critical care nurses in Europe and the United States failed to answer even the most basic questions concerning the PAC and its measurements [1, 2]. The aim of this narrative review is to provide an overview of the existing knowledge on the use of the contemporary PAC in critically ill and perioperative patients. This CCO-PAC, further mentioned as PAC, is a 7.5 F continuous cardiac output (CCO)/mixed venous oxygen saturation (SvO_2_)/ end diastolic volume (EDV)-pulmonary artery catheter (model 774F75; Edwards Lifesciences, Irvine, CA, USA). In the first part of this review we discussed adequate placement, interpretation of waveforms, as well as pitfalls of this PAC. In this second part of the review, we highlight measurements of the additional information that comes from the technological innovations of the contemporary PAC, including the measurement of CCO, RV ejection fraction (RVEF), end-diastolic volume index (EDVi), and SvO_2_. Limitations and clinical applications of these measurements are addressed in detail.

## Measurements

Measurements obtained from the PAC can be found in Table 1. It is of note that for accurate measurements the PAC should be placed in the correct position within the pulmonary artery. This procedure is described in detail in the first part of this review [3].

**Table 1:** Hemodynamic variables obtained from the pulmonary artery catheter

Variable	Abbreviation	Equation	Normal range
Mixed venous oxygen saturation	SvO_2_	n.a	60–80%
Cardiac output	CO	HR × SV/1000	4.0–8.0 L min^–1^
Cardiac index	CI	CO/BSA	2.5–4.0 L min^–1^ m^−2^
Cardiac power index	CPI	(MAP-CVP) × CI/451	0.5–0.7 W m^−2^, population specific
Central venous Pressure	CVP	n.a	2–6 mmHg
Stroke volume	SV	CO/HR × 1000	60–100 mL
Stroke volume Index	SVi	CI/HR × 1000	33–47 mL m^−2^
Stroke volume variation	SVV	(SVmax-SVmin)/SVmean × 100	10–15%
Systemic vascular resistance	SVR	80 × (MAP − CVP)/CO	800–1200 dynes sec cm^–5^
Systemic to pulmonary pressure ratio	MAP/MPAP	MAP / MPAP	4.0 ± 1.4 in uncomplicated cardiac surgey
Pulmonary artery systolic pressure	PASP	n.a	15–30 mmHg
Pulmonary artery diastolic pressure	PADP	n.a	8–15 mmHg
Pulmonary artery wedge pressue	PAWP	n.a	6–12 mmHg
Pulmonary vascular resistance	PVR	80 × (MPAP − PAWP)/CO	< 250 dynes sec cm^−5^
Pulmonary artery pulsatility index	PAPI	(PASP − PADP)/CVP	population specific
LV stroke work index	LVSWi	SVi × (MAP − PAWP) × 0.0136	50–62 mmHg ml m^−2^
RV stroke work index	RVSWi	SVi × (MPAP − CVP) × 0.0136	5–10 mmHg ml m^−2^
RV function index	RFI	PASP/CI	31.7 ± 16.7 in ICU survivors with PH
RV end-diastolic volume	RVEDV	SV/EF	100–160 mL
RV end-diastolic volume index	RVEDVi	RVEDV/BSA	60–100 mL m^−2^
RV end-systolic volume	RVESV	EDV-SV	50–100 mL
RV ejection fraction	RVEF	(SV/EDV) × 100	40–60%
RV systolic pressure	RVSP	n.a	15–30 mmHg
RV diastolic pressure	RVDP	n.a	2–8 mmHg

*BSA* body suface area; *CI* cardiac index; *EDV* end diastolic volume; *EF* ejection fraction; *HR* heart rate; *LV* left ventricle; *MAP* mean arterial pressure; *MPAP* mean pulmonary arterial pressure; *n.a.* not applicable; *PAWP* pulmonary artery wedge pressure; *PH* pulmonary hypertension; *RV* right ventricle

Adapted from: Edwards Clinicical Education Quick Guide to Cardiopulmonary Care [4]

## Cardiac output

### Intermittent cardiac output measurements

The Fick method is the gold standard for indirect CO determinations. This method determines the cardiac output as the quotient of systemic oxygen consumption (VO_2_) and the difference between arterial and mixed venous oxygen content.

The oxygen concentration in arterial blood is a function of the hemoglobin concentration (Hb) and the percent saturation of hemoglobin with oxygen (SaO_2_). The CO can then be calculated using the following formula:

$$-CO \, (mL\min)^{-1} = \frac{{VO_{2} }}{{1.34 x Hb x \left( {SaO_{2 }- SvO_{2} } \right)}}$$

In this formula, VO_2_ (in mL min^−1^) = oxygen consumption as directly measured by respirometry [5], SvO_2_ (in %) is the mixed venous oxygen saturation. Since this direct Fick technique is technically demanding at the bedside, it is rarely used in clinical practice. 

Intermittent pulmonary artery thermodilution is the clinical reference method for CO measurement [6]. To measure CO using pulmonary thermodilution, a bolus of cold crystalloid solution is injected in the central venous circulation. The cold indicator bolus injection causes a decrease in blood temperature that is detected downstream using a thermistor near the catheter tip. From the thermodilution curve, which represents the changes in blood temperature over time, CO can be calculated using a modified Stewart-Hamilton formula:

$$CO=\frac{V x \left(Tb-Ti\right)}{A} x\frac{SI x CI}{(SB x CB)}x\frac{60 x CT x K}{1}$$

In this formula, CO = cardiac output, V = volume of injectate, A = area of thermodilution curve in square mm divided by paper speed (mm/sec), K = calibration constant in mm/˚C, Tb = temperature of blood, Ti = temperature of injectate, SB = specific gravity of blood, SI = specific gravity of injectate, CB = specific heat of blood, CI specific heat of injectate,

$$\frac{SI x CI}{(SB x CB)} = 1.08$$

when 5% dextrose is used, CT is correction factor for injectate warming.

Intermittent pulmonary artery thermodilution with cold-saline bolus injections has multiple limitations. The modified Steward-Hamilton equation shows that the bolus-derived information depends on injected volume, rate, and temperature. Overestimation of CO can occur in the presence of left-to-right or right-to-left intracardiac shunts, the use of a small injection volume, or a higher injectate temperature as compared to the reference temperature. All of these causes result in a smaller area under the thermodilution curve, resulting in an overestimated CO. Tricuspid regurgitation (TR) might both under- and overestimate CO due to increased transit time and modified blood temperature in the right atrium. Pulmonary valve insufficiency changes the appearance of the thermodilution curve, but CO measurement generally remains accurate since the area under the thermodilution curve is not affected, unless the CO is very low [7]. Underestimation of CO is mainly seen in high-flow states due to rapid temperature changes in the pulmonary artery [8–11]. In addition, inadequate timing during the respiratory cycle and variability in injection technique may further influence the accuracy of bolus thermodilution CO measurements [12]. Bolus CO measurements are therefore highly user-dependent [13]. Over the years a continuous measurement system has been developed in order to overcome these disadvantages. In the early days, placement of a heating filament was severely compromised due to background thermal noise in the pulmonary artery or because of limitations either in maximum peak heat flux or in temperatures [14, 15]. To overcome these limitations, a combination of thermal indicator dilution and a stochastic system is now used in the modern PAC. To this end, the contemporary PAC is equipped with a 10 cm long thermal filament, positioned 15–25 cm from the tip of the catheter. This filament heats up the blood in a random on–off pattern. The change in blood temperature is measured downstream by the thermistor throughout the entire respiratory cycle. Based on a repeating on–off signal, a relaxation waveform can be generated. This technique enables measurement of true volumetric flow and is independent of the physical geometry of the system. Detailed information about the used algorithm and the stochastic system has been described previously [16].

### Continuous cardiac output measurement

**Fig. 1:**
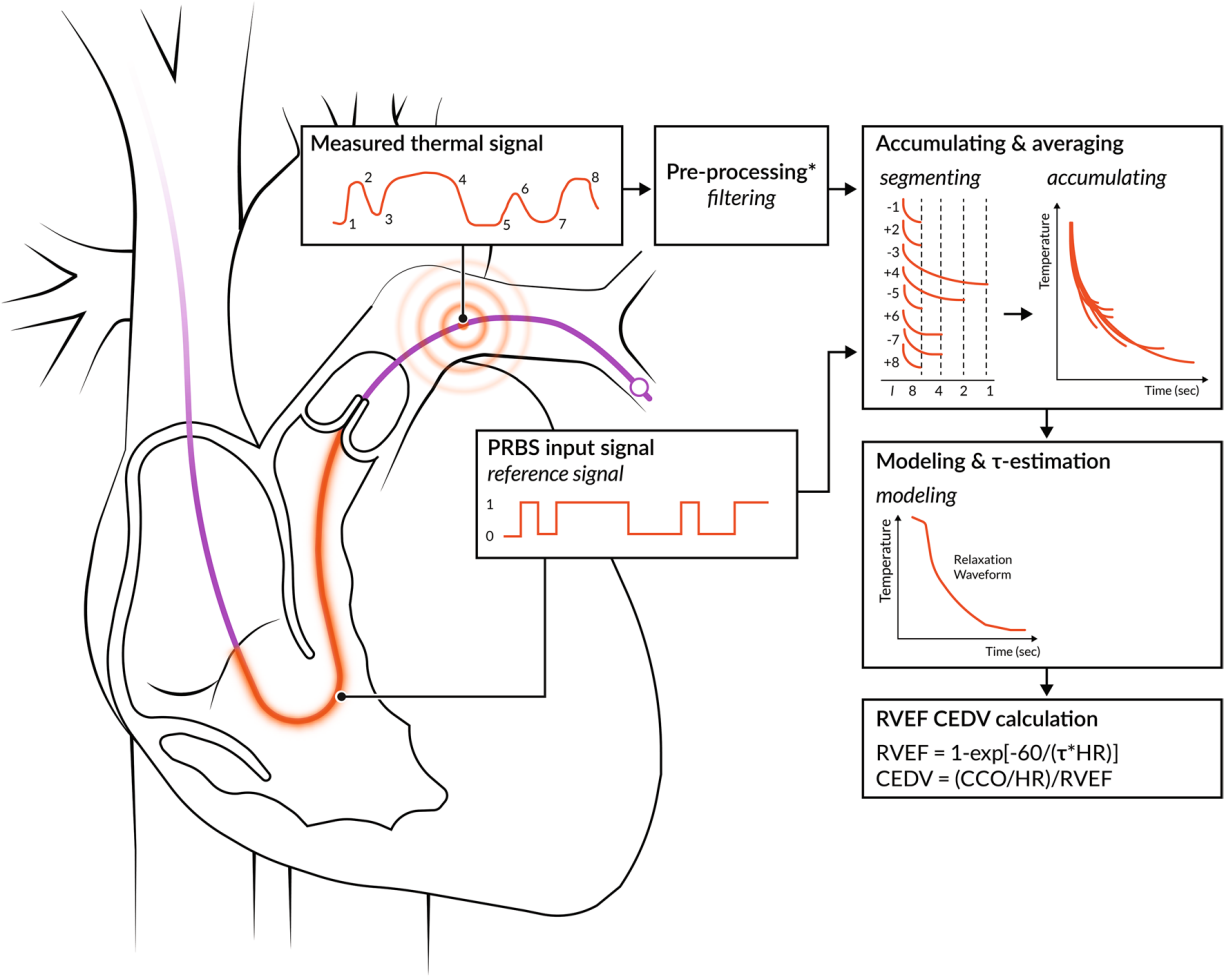
Relaxation waveform for continuous cardiac output and concomitant calculations of right ventricular ejection fraction and right ventricular end-diastolic volume calculations. Shown are the thermal signal sent out by the proximal part of the PAC, how this is received in the more distal part of the PAC, and how this is transformed to derive the specific variables. PRBS Pseudo-Random Binary Sequence; RVEF right ventricular ejection fraction. CEDV continuous right ventricular end-diastolic volume. CCO continuous cardiac output. τ = exponential decay time constant. * This step is skipped when using STAT-CCO over trend CCO monitoring. Adapted from: Wiesenack C et al. [46]

Using the area under the relaxation thermodilution waveform, near-continuous and almost real-time measurement of CCO can be obtained. CCO measurement with PAC is well-validated in experimental settings nowadays, as well as in different patient categories [17–20]. CCO was shown to be more accurate when compared to various other measurement methods for CO, including electromagnetic measurement of aortic blood flow, bolus thermodilution, the Fick method, and aortic transit-time ultrasound [18, 21–25]. In addition, CCO showed to be more accurate and less variable when compared to the intermittent bolus thermodilution technique. The CCO method is independent of the clinician, injection technique, and injection volume. Furthermore, the CCO method is not influenced by ventilator settings due to a high sampling rate at random time points in the ventilatory cycle (Fig. 1). This allows for detection of smaller variations in CO, as well as good performance over a wide range of CO and blood temperatures [24, 26].

## Limitations of cardiac output measurements

### Delayed response in CCO measurement results

It is important to distinguish two different ways of depicting CCO measurement results: trend CCO and STAT CCO. The trend CCO reflects the average CCO over the previous 4–12 min (depending on the monitor setting) [27, 28]. During rapid alterations of hemodynamic state there is a clinically important time lag in the response of the trend CCO [28]. The STAT CCO was designed to improve the response time. Using a faster algorithm, STAT CCO is updated every 30–60 s and has shown good accuracy and precision compared to intermittent pulmonary artery thermodilution [28]. Pacing-induced hemodynamic changes, for instance, were detected in mean arterial pressure (MAP) recordings after 30 s, and an increase in SvO_2_ reached significance after 90 s. A significant increase in CCO using the STAT algorithm was reached after a minimum of 270 s [28]. Physicians should be aware of a delayed response, even when using the STAT mode [29, 30], limiting the use of this method in dynamic hemodynamic assessments (e.g. of fluid responsiveness).

### Intracardiac shunts

In vitro experiments have shown that shunting 50% of total blood flow results in mean systematic errors of − 26.8 (± 8.2%) for CCO measurements during an intracardiac left-to-right shunt, overestimating true values as a result of altered waveform configuration [24]. Although the CO is falsely high in the setting of intracardiac shunts, the PAC may be useful in both detecting the presence and assessing the magnitude of the intracardiac left-to-right shunt.

### Tricuspid regurgitation and tachycardia

TR has been associated with an underestimation or overestimation of CO, and even with no influence on CO measurements [31, 32]. In general, a high degree of TR is associated with an underestimation of true CO [33]. In patients with pulmonary hypertension, the agreement between the Fick method and thermodilution CO was not affected by the severity of TR [34]. However, despite possible under- or overestimation of CCO in the presence of TR, CCO measurements remain clinically relevant when using this method for trend monitoring, as well as to assess the response to hemodynamic interventions [35]. Furthermore, when using the CCO method, it might be expected that the influence of TR is less pronounced when compared with the intermittent bolus CO technique, because CCO represents an average value over time and is less dependent on interindividual variations in infusion. However, firm data on this remain scarce [26].

### Fluid administration

The infusion of fluid through the side-ports of the sheath, or through the venous port of the PAC, influences the thermodilution washout curve. During bolus thermodilution measurements this leads to an artefactual increase in the area under the curve, and thus to an underestimation of CO [36, 37]. Although it is suggested that the continuous measurement system is less accurate during fluid infusion [38], various infusion rates of lactated Ringer’s solution (100, 200, 500, 1000 ml h^−1^) only influenced the CCO values at a low-flow rate equal to or below 2 L min^−1^. In contrast, intermittent bolus CO measurements were affected at all flow rates. Thus, CCO measurements seem to have a better resistance to the thermal noise produced by high rates of infusions as compared to the bolus method [24].

### Extreme temperature variations

Extreme temperature variations can cause a poor correlation between intermittent bolus CO measurements and CCO measurements. In patients treated with therapeutic hypothermia after cardiac arrest, for instance, a low correlation coefficient was observed with broad limits of agreement when comparing thermodilution CCO with indirect Fick CO measurements [39]. Conflicting results were found in small, non-randomized trials in the setting of post cardiopulmonary bypass [40, 41]. In patients undergoing orthotopic liver transplantation, both CCO and bolus CO methods showed decreased accuracy and precision after caval clamping and reperfusion [20]. However, since the accuracy of bolus CO among hypothermic patients is a topic of debate, this method may not necessarily be considered the standard for comparison in this specific setting. It is of note that in vitro measurements indicate that the CCO technique has a greater resistance to thermal noise compared to bolus CO measurements providing a higher accuracy [24].

## Right ventricular ejection fraction and end-diastolic volume

At the end of the 1980′s, a PAC enabling measurement of both RVEF and right ventricular end-diastolic volume (RVEDV) was introduced. This PAC was validated against various other RVEF measurements methods, including angiography, contrast ventriculography, and echocardiography [42–44]. Nowadays, the PAC enables continuous measurement of the RVEF and RVEDV by using the exponential decay time constant (τ) of the thermodilution relaxation waveform, combined with the heart rate (HR) (Fig. 1). RVEF is calculated as follows:

$$RVEF = 1 - \exp \frac{ - 60}{{\tau xHr}}\,or\,RVEF = 1 - \frac{Tb2 - Tb0}{{Tb1 - Tb0}}$$

where Tb0 = blood temperature before heat application, Tb1 = blood temperature during the first subsequent systole, and Tb2 = blood temperature during the successive systole [45]. Once RVEF is obtained, calculations of RVEDV are based on CCO, HR, and RVEF using the following calculation [46]:

$$RVEDV=\frac{\left(CCO/HR\right)}{RVEF}.$$

Because RVEDV is derived from RVEF and CO, errors in RVEDV are a combination of errors in both CO and RVEF measurements. Nevertheless, RVEDV has been proven to be highly predictive for volumes in a pulsatile flow model [47]. Measurements of RVEF and RVEDV are neither dependent on bolus volume nor on the temperature of the injected fluid. Moreover, in CCO, the PAC filaments heat up the blood directly in the RV, bypassing the influential effects of the right atrium, leaving the conservation of energy and RV dynamics and RV afterload as the primary determinants of the thermal washout curve [48]. As a result, previous studies using the bolus thermodilution technique may not directly be comparable with the CCO approach using the heating filament. Although the continuous thermodilution technique is currently validated for CCO measurements, there is lack of data concerning validation of RVEF and RVEDV. Overall, both reproducibility and accuracy of the continuous method are superior compared to the intermittent bolus technique [24, 26]. Since the RVEF uses the washout thermodilution curve, all factors that confound CO measurements will also interfere with an accurate determination of RVEF.

### Limitations of the continuous RVEF measurements: Underestimation

In general, every measurement method has its own unique reference values. Thermodilution-derived RVEF seems to underestimate RVEF when compared to other measurement methods such as ultrasound, magnetic resonance imaging (MRI), and radionuclide angiography [42, 43, 45, 49–51]. Animal research revealed that this underestimation was most likely explained by the fact that the blood in the right atrium did not return to baseline temperature within a single heartbeat after the cold fluid injection [48]. Although thermodilution with the continuous measurement technique takes place in the RV instead of the right atrium, the continuous RVEF still seems to be underestimated by the PAC [45]. New 4D MRI technology has revealed that the blood temperature did not return to baseline within a single heartbeat as a result of the phasic contraction pattern of the RV. For every systolic beat, only 44% of the EDV contributed directly to the pulmonary artery flow [52]. Recirculation of blood in the RV might result in it taking more time for the heat mass to reach the thermistor. As a consequence of the prolonged relaxation, waveform RVEF will be underestimated and RVEDV will be overestimated. Whether or not the absolute volume data is completely correct does not influence whether these measurements are precise, and the fact remains that they can be of great value for trend monitoring. In general, an absolute correction factor of + 11% will result in a more realistic absolute value of RVEF [48].

### Mathematical coupling

Since the formula of RVEDV contains the CO by dividing stroke volume (SV) by RVEF, the correlation between those two variables may be explained by mathematical coupling. However, various studies examining the relationship between RVEDV and CCO showed that this relationship remained significant even after statistical correction for potential mathematical coupling or by including an independent technique for CO measurements. Therefore, mathematical coupling alone does not explain the correlation between RVEDV and CO [53–55].

## Mixed venous oxygen saturation

Mixed venous oxygen saturation (SvO_2_) can be measured periodically in blood samples drawn from the distal lumen of the PAC in order to validate the measured values. Adding reflective fibreoptic oximetry at the distal end of the PAC enabled the clinician to accurately measure the SvO_2_ on a continuous base [56]. Oximetry is based on the technique known as spectrophotometry. The absorption of specific wavelengths of light, as it passes through a medium, is proportional to the concentration of the substance that absorbs both the light waves and the travel length. Oxygenated Hb does not absorb red light waves (wavelength 660 nm) as well as deoxygenated Hb does. On the contrary, infrared light waves (wavelength 940 nm) are better absorbed by oxygenated Hb. The determinants of SvO_2_ are identified in the following equation:

$$\text{SvO}_2=\text{SaO}_2 - (\text{VO}_2{\text {CO}}\times 1.34\times [\text{HB}]),$$

where SaO_2_ is arterial oxygen saturation and VO_2_ is systemic oxygen consumption. As such, SvO_2_ reflects the balance between oxygen delivery (DO_2_) and oxygen consumption (VO_2_). A change in SvO_2_ indicates an imbalance between oxygen delivery and consumption. However, further information is needed to assess the cause of this change. Therefore, SvO_2_ is not a simplified index of inadequate CO, since there are more determinants in the formula. Alterations in SvO_2_ might be due to changes in oxygen transport (arterial SaO_2_, Hb, CO) or a change in body VO_2_ [5].

## Clinical application of PAC-derived data

### Assessing fluid responsiveness

Over the years it has become clear that static filling pressures (CVP and PAWP) and cardiac preload should not be used interchangeably [57–59]. The pressure–volume relationship of the RV has a triangular shape, due to the low pressure and high capacitance characteristics of the pulmonary vascular bed. The RV pressure–volume relationship changes with different loading conditions, which can result in an increased filling pressure associated with a decreased filling volume [60, 61]. A change in preload does not result in a proportional change in filling pressures [62]. Although CVP and PAWP are not suitable for preload assessment, this does not mean that they should not be measured at all. An important determinant of organ perfusion pressure is the difference between the inflow pressure (MAP) and the outflow pressure (CVP). Both lower MAP and elevated CVP can result in diminished organ perfusion. Among different patient categories, an association between elevated CVP and impaired microcirculatory blood flow or increased risk of acute kidney and liver injury has been demonstrated [63–65]. Elevated or rapidly rising values of CVP and PAWP may serve as a stop rule for fluid resuscitation [66]. An increase in CVP in response to a fluid challenge without a change in CO is an indicator for poor fluid responsiveness and should alert the clinician of a possible RV dysfunction [66, 67]. The work of Guyton showed how venous return curves interact with cardiac function curves, i.e. right atrial pressure not being the primary determinant of CO rather than itself being determined by CO [68, 69]. When combining this knowledge with blood pressure difference (MAP-CVP) and CO, clinicians are offered a potential approach regarding the application of CVP in the clinical setting (Table 2) [70]. Although it has been shown that many intensive care physicians do not measure CO, it is highly recommended when trying to obtain a better understanding of both the hemodynamic situation and the effects of goal-directed management [71].

**Table 2:** PAC-derived variables in the clinical setting

Clinical situation	PAC derived variables	Clinical interpretation
Low arterial blood pressure	↓ CCI + ↑ CVP	Decrease in venous return, e.g. reduced cardiac function or hypovolemia
	↑ CCI + ↓ CVP	Increase in venous return, e.g. distributive shock
Fluid responsiveness	↑ SV or CCI ≥ 15% after 250 ml or 3 ml kg^−1^ of crystalloid	Patient will probably benefit from fluid administration
RV dysfunction and failure	Early stage (moderate RV dysfunction = RVEF 20–30%): ↓ RVEF, ↑ EDVi, CCI = N, SvO_2_ = N CVP N or ↑	
	Advanced stage (severe RV dysfunction = RVEF < 20%): ↓↓ RVEF, ↑ EDVi, ↓ CCI, ↓ SvO_2_, ↑ CVP	
LV failure	↑ PAP, ↑ PAWP, ↓ CCI	
Intracardiac shunt	↑↑ SvO_2_	≥ 6% step up ScvO_2_ to SvO_2_ indicates a L-R shunt
Weaning- from-ventilator	↑ PAWP, ↓ SvO_2_ during weaning trial	Weaning-induced cardiac failure
Pulmonary hypertension	Pre-capillary: PAWP =N Post-capillary: ↑ PAWP (> 15 mmHg)	Echocardiographic assesment should rule out HFpEF
Tamponade post cardiac surgery*	↓ CCI, ↑/ = CVP, ↓ SvO_2_, ↓ EDVi, ↓ RVEF, ↑ PAWP	Compression of the RV free wall causes low RVEDV despite substantial fluid administration
Distributive shock	↑ CCI, ↑ SvO_2_, ↓/ = CVP, ↓/ = PAWP	Septic, neurogenic, anaphylactic, toxin-induced, or endocrine shock
Hypovolemic shock	↓ CCI, ↓ SvO_2_, ↓CVP, ↓ PAWP	Hemorrhagic, gastrointestinal, skin, renal, or third space fluid losses
Cardiogenic shock	↓ CCI, ↓ SvO_2_, ↑/ = CVP, ↑ PAWP	Cardiomyopathic, arrhythmic, or mechanical causes
Obstructive shock	↓ CCI, ↓ SvO_2_, ↑ CVP, ↑ PAWP	PH, pulmonary embolism, tension pneumothorax, tamponade, pericarditis, restrictive cardiomyopathy

^*^The location of the bleeding/hematoma determines the hemodynamic profile of the patient

*N* normal; *CCI* continuous cardiac index; *CVP* central venous pressure; *SV* stroke volume; *RV* right ventricle; *RVEF* right ventricular ejection fraction; *EDVi* end-diastolic volume index; *SvO*_*2*_ mixed venous oxygen saturation; *ScvO*_*2*_ central venous oxygen saturation; *PAP* pulmonary artery pressure; *PAWP* pulmonary artery wedge pressure; *LV* left ventricle; *HFpEF* heart failure with preserved ejection fraction; *PH* pulmonary hypertension; *HFpEF* heart failure with preserved ejection fraction

Today, static filling pressures are replaced by the concept of fluid responsiveness. The Frank-Starling curve depicts SV on the vertical axis and cardiac preload on the horizontal axis. On the steep part of the curve, an increase in preload will result in a significant increase in SV. At higher values of cardiac filling pressures, the curve flattens and an increase in preload will not result in an increase in SV [72]. In this respect there are three relevant questions in the clinical setting: (1) At which part of the Frank-Starling curve does the heart of the patient operate? (2) Is the patient fluid responsive? (3) Are fluids beneficial? Irrespective of the answers to question 1 and 2, the clinician does need to determine whether fluids are beneficial to the patient, or whether another therapeutic approach is needed or better suited to the situation, since being fluid responsive is not equivalent to being in need for fluids. Fluid challenges should be performed with 250 ml or 3 ml kg^−1^ crystalloid, which is infused over a short period of time (5–10 min). Fluid responsiveness is most often defined as an increase ≥ 15% in stroke volume index (SVi) or cardiac index (CI) after a fluid challenge (Table 2) [73]. SVi or CI should be the primary target, and neither arterial blood pressure nor ventricular filling pressures or volumes should be used as a surrogate for fluid responsiveness [74]. CI and SVi measured with the PAC are highly predictive of actual pulsatile flow [47]. Since the PAC is able to measure both fluid responsiveness variables (SVi and CI) and target/safety thresholds (CVP and PAWP) in a continuous manner, it can be used to manage fluid therapy adequately [29, 30]. In addition, a rise in RVEDP during fluid administration, in the absence of a change in CO, is indicative for RV volume overloading and a reason for the clinician to stop the intervention.

### Right ventricular dysfunction and failure

Acute RV dysfunction can occur due to a variety of diseases, resulting in an increase in RV afterload, decreased contractility, or an increase/decrease in RV preload. A decreased RV function can induce a vicious circle of RV failure. When having a closer look at hemodynamics during RV failure, ventricular interdependence is an important concept to keep in mind. Due to shared muscle fibers, septal wall, and pericardium, mechanical forces can be transmitted from one ventricle to the other, both in systole and diastole [75]. RV volume/pressure overload or diminished contractility will result in RV dilatation. The intraventricular septum will flatten during diastole in case of volume overload and mainly during systole in case of pressure overload, creating a D-shaped LV [75, 76]. RV diastolic dysfunction and RV dilatation will shift the pressure–volume curve of the LV towards higher pressures, due to decreased LV diastolic compliance [75]. Furthermore, increased LV end-diastolic pressure (LVEDP), reduced LV transmural filling pressure, and impaired LV diastolic filling as a result of the septal shift will contribute to low CO and ultimately to low blood pressure [77]. In severe RV failure, low blood pressure in combination with high RV filling pressures result in severely reduced organ perfusion, due to a reduced difference between MAP and CVP, being an important determinant of the driving force for venous return [78]. It is of note that a normal CO, or normal pulmonary artery pressure (PAP), does not exclude RV dysfunction [79, 80]. Classically, the diagnosis of RV failure is made by combination of clinical assessment (i.e. signs of impaired organ perfusion in combination with venous congestion) and echocardiographic evaluation. To classify RV failure, a number of reference values for a variety of echocardiographic measures have been suggested [81]. Providing RV volume and pressures with the PAC, as well as contractility measurements, can be helpful in diagnosing and managing RV failure. In Table 1 reference values for RVEF and RVEDV have been provided, as stated by the manufacturer. However, it is pivotal to understand that reference values for PAC-derived RVEF in the clinical setting may be considerably lower, also in comparison to 2D or 3D echocardiography. Based on datasets, combining RVEF with long-term outcome in cardiac surgery and sepsis, we suggest the following classification: RVEF < 20%: severe RV dysfunction; RVEF 20–30%: moderate RV dysfunction: RVEF > 30%: no RV dysfunction [82, 83]. Under physiological conditions, an increase in RVEDV is compensated by an (immediate) increase in SV, referred to as heterometric autoregulation [84]. However, in the early stage of RV dysfunction, RV dilatation becomes an adaptive mechanism for the preservation of adequate preload, reflected by a higher EDV and lower RVEF. When RV failure is combined with, or secondary to, LV failure, PAWP can be elevated (Table 2). In a more progressive disease state, CO will be diminished as well. The CVP waveform can reveal a prominent v-wave due to TR in response to RV dilatation [85].

Nowadays, new hemodynamic indices, derived from PAC measurements, might be helpful in early identification of RV dysfunction. The pulmonary artery pulsatility index (PAPi) is defined as: (systolic PAP − diastolic PAP) / CVP. This index predicts severe RV failure and has additive value in the setting of advanced heart failure, cardiogenic shock, and left ventricular assist device therapy. However, PAPi measurements and thresholds vary significantly between studies of different patient populations and thresholds from one patient population should not be extrapolated to another patient group [86].

Another index is the ratio of pulmonary artery effective elastance (E_a_) to RV maximal end-systolic elastance (E_max_). This right ventriculo-arterial coupling index relates to the mechanical efficiency of the RV, and is ideally derived from RV pressure–volume loops. Nowadays, bedside estimation can be obtained by this ratio, using the contemporary PAC. E_a_ and E_max_ can be defined as E_a_ = mean PAP

$$\begin {aligned} &{\text{MPAP}}-{\text{PAWP}}/ {\text{SV,}} \\ &{\text{and E}}_{\text{max}} ={\text{MPAP}} /  {\text{RVEDV}}-{\text {SV}}. \end {aligned}$$

The ratio E_a_/E_max_ equals 1 in case of optimal ventricular-vascular coupling. Hence, E_a_/E_max_ may help in early identification of RV dysfunction in critically ill patients [87].

Under conditions of impaired RV function, analysis of the RV waveform can be useful in early detection and subsequent management of RV dysfunction, especially during cardiac surgery [88–90]. Since RV pressure monitoring requires a different PAC with a dedicated RV pace-port, further details are beyond the scope of this review.

### Left heart failure

To distinguish isolated RV failure from a combination of RV and LV failure, the use of PAC may be helpful. Of note, LV filling pressures cannot be reliably estimated by means of clinical examination [91]. Classically, in case of combined LV and RV failure, CI and SvO_2_ are low, and PAWP is elevated [92]. In patients with a PAWP ≥ 15 mmHg, LV failure is likely [93]. In case of a low or normal PAWP, isolated RV failure is more likely. However, a PAWP ≤ 15 mmHg does not rule out the presence of LV failure, in particular in patients with LV heart failure and preserved ejection fraction (HFpEF) [94]. In this case, further echocardiographic evaluation of diastolic LV function is recommended (Table 2).

### The detection of left-to-right shunts

A high SvO_2_ > 75% may indicate a cardiac left-to-right shunt. For oximetric shunt detection, blood sampling from the distal channel in the PAC and the proximal channel in the vena cava superior or right atrium is needed. Under physiological conditions, oxygen saturation in the pulmonary artery is lower than that in a central vein, due to the contribution of desaturated blood flow from the coronary sinus. However, when a left-to-right shunt is present, oxygenated blood can cause an increase in oxygen saturation at the tip of the PAC. A step up of > 6% in oxygen saturation from the vena cava superior to the pulmonary artery is suggestive of the presence of a left-to-right shunt (Table 2) [95]. Using the SvO_2_ and the central venous saturation (ScvO_2_) in combination with the arterial oxygen saturation (SaO_2_), a shunt fraction can be calculated according to the following equation:

$$\text{Qp/Qs}=(\text{SaO}_2 - (\text{SvO}/{\text {SpvO}}_2) /({\text{SpvO}_2}-{\text{SpaO}_2}),$$

where Qp = pulmonary blood flow, Qs = systemic blood flow, SaO_2_ = arterial oxygen saturation, SvO_2_ = central venous oxygen saturation, SpvO_2_ = pulmonary vein oxygen saturation (in the absence of a right to left shunt, this is identical to SaO_2_), and SpaO_2_ = pulmonary artery oxygen saturation [95].

### Ventilator weaning-induced cardiac failure

When switching from positive pressure ventilation (with and without positive end-expiratory pressure; PEEP) to spontaneous breathing, intrathoracic pressure falls during both inspiration and expiration compared to assisted ventilation. In response, right atrial pressure falls and venous return increases, resulting in an increase in RV preload, an increase in CO (in the fluid responsive patient), and in LV preload. In addition, the negative intrathoracic pressure results in an increase in LV afterload [96]. Besides these pressure changes, hypoxemia, hypercapnia, and an increased sympathetic tone can result in an increase of RV or LV afterload. However, in a specific subgroup of patients, right atrial pressure may rise during a spontaneous breathing trial [97]. This might be explained by an increase in intrinsic PEEP due to expiratory muscle activity or dynamic hyperinflation [98, 99]. When following this line of thought regarding physiology, one can see that an elevated PAWP can be the result of an increase in LV preload in patients with an already elevated LV end diastolic volume (LVEDV), an increase in afterload, for example due to a subsequent increase in mitral insufficiency, or a decrease in LV compliance (or a combination of these). In a landmark paper [100], elevated PAWP (> 18 mmHg) during a spontaneous breathing trial was shown to be associated with subsequent weaning failure in patients diagnosed with severe chronic obstructive pulmonary disease. After restarting mechanical ventilation, all patients received diuretics, and the PAWP decreased markedly as compared to before treatment (9 vs. 25 mmHg). In addition, failure to wean the patient from the ventilator was also accompanied by a decrease in PAC-derived SvO_2_ measurements, whereas SvO_2_ remained unchanged in the successfully weaned patients. The same study revealed no change in CI combined with an elevation of PAP and PAWP, indicating an increase in both LV and RV afterload [101]. PAC measurements can reveal weaning-induced cardiac failure, showing the response of the RV and LV during spontaneous breathing, as well as providing information about the change in the VO_2_/DO_2_ balance during this critical period. In daily practice, PAWP should be measured before and after a 30 min spontaneous breathing trial [102]. A T-piece weaning trial challenges patients’ efforts and the LV performance the most. Other ways of conducting weaning trials, such as applying low levels of pressure support ventilation, might not reveal an elevation in PAWP (Table 2) [103].

### Pulmonary hypertension

Right heart catheterization is the diagnostic gold standard for assessing pulmonary hypertension (PH), which was classically defined as a MPAP ≥ 25 mmHg at rest, and recently updated to a MPAP > 20 mmHg at rest [104–106]. In patients with high MPAP, PAWP ≤ 15 mmHg is used to distinguish pre-capillary PH from high PAP pressures due to LV failure, since higher wedge pressures are related to left heart disease (Table 2) [93]. However, PAWP ≤ 15 mmHg does not rule out the presence of left heart failure, in particular in patients with HFpEF [94]. Relying on a single measurement can falsely label patients with an inaccurate diagnosis. In order to distinguish precapillary PH from HFpEF, additional echocardiographic assessment in combination with the assessment of risk factors associated with HFpEF may avoid misclassification [104]. Once the suspicion of PH has risen because of high PAP measurements, it is recommended to refer patients to an expert PH centre for further diagnosis and treatment early in the diagnostic process [107]. In the ICU, PH is rarely the primary cause of admission so that clinicians should search for underlying disease states that cause PH; however, exact data remain scarce [108]. Upon hospital admission, high PAP values are mostly seen as secondary to acute conditions, such as pulmonary embolism, acute respiratory distress syndrome, LV failure, or mitral valve regurgitation [109]. The classification of chronic PH is not always applicable in critical care settings and a different classification according to the underlying cause has been suggested [110]. Since the RV is not resistant to acute increases in afterload, acute PH can result in RV failure [111, 112].

### Restrictive pathophysiology and tamponade

Pericardial constriction, restrictive cardiomyopathy, and RV infarction share the same underlying pathophysiologic feature; reduced RV diastolic compliance due to an increase in RV stiffness or impaired RV relaxation [113]. CVP waveform analysis can provide additional diagnostic clues for these conditions. Cardiac tamponade can be distinguished by the attenuation or disappearance of the y-descent in the CVP waveform. Obstructive shock due to tamponade results in a low CO, low SV, low MAP, and high CVP and RV filling pressures. Pulsus paradoxus can be present. In the final stage, there will be an equilibration of all cardiac and pulmonary artery diastolic pressures, which will result in an absence of coronary flow. This will finally lead to a circulatory arrest (Table 2) [114].

However, in the setting of postoperative cardiac surgery, the above described classical forms of waveforms and hemodynamic patterns may not be present during tamponade. The specific location of well-defined hematomas, rather than free mobile accumulation of fluid, determines the specific combination of alterations in waveforms, pressures, and volumes. For example, compression of the RV free wall by a localized hematoma may cause low RVEDV and low continuous cardiac index (CCI), despite substantial fluid administration, in combination with elevated or normal CVP (Table 2).

### Determination of shock type

In shock, there is a mismatch between systemic oxygen delivery and oxygen demand. There are four types of shock; hypovolemic, cardiogenic, obstructive, or distributive. The PAC can be useful in identifying the type of shock, and it can be beneficial during the assessment of the hemodynamic status, as a prerequisite to select the adequate therapeutic intervention, and to evaluate the response to therapy. In current guidelines, if clinical examination alone does not lead to a diagnosis, use of the PAC is recommended in complex patients for the determination of the type of shock, in patients with refractory shock, and for shock in combination with RV dysfunction or acute respiratory distress syndrome [74, 115].

### An integrative approach

Combining various variables may help to further elucidate the underlying mechanisms of RV failure, and strives beyond the strict interpretation of references values. For example, at first glance PAP values may not seem too far above the threshold for PH. But in case systemic blood pressure is below normal at the same time, such value may gain additional importance. The systemic to pulmonary pressure ratio (MAP/MPAP) is a tool to quantify such ‘relative’ PH and appeared useful in the prediction of hemodynamic complications during and after cardiac surgery [116]. Adjusting the PAP for a specific CI helps to quantify the RV workload, which is needed to maintain RV performance in the presence of a given afterload. The RV function index (RFI), defined as the systolic PAP(SPAP)/CI ratio, may be helpful to assess the additional amount of effort for the RV in case the flow or the afterload increases, and has predictive value as an independent risk factor for mortality in ICU patients with PH [117]*.* Finally, integrating the driving pressure (MAP-CVP) with the flow (CI), by means of a cardiac power output (CPO), elegantly acknowledges the fact that maintenance of the CI within the normal range, at the expense of an elevated CVP is less energy effective than maintaining an equal CI in the presence of a normal CVP [118]. As such, the CPO may be helpful to guide hemodynamic therapy into an acceptable range of MAP and CI, at the lowest possible level of VO_2_.

## Complications of the PAC

The invasive nature of the PAC implies the risk of complications. First of all, central venous access can result in accidental arterial puncture, air embolism, and pneumothorax. However, using ultrasound guidance during placement has been demonstrated to reduce the risk of catheter misplacement [119–121]. Secondly, several complications can arise due to the catheterization itself, such as severe dysrhythmias, right bundle branch block, or complete heart block. Minor dysrhythmias occur often during catheter insertion or withdrawal but resolve spontaneously after advancing the catheter through the RV [120]. Lastly, prolonged catheter residence can result in pulmonary artery rupture, pulmonary infarction, or venous thrombosis [6]. Catheter-related infections with the PAC are uncommon and involve the introducer sheath rather than the PAC itself [122]. Increased infection risks are associated with prolonged PAC use, insertion via the internal jugular vein rather than the subclavian vein, and unsterile procedures [122, 123]. Right heart catheterizations performed in experienced centres are associated with low risk of serious complications, and there is high quality evidence that PAC use does not alter mortality [6, 124]. Absolute contraindications of PAC placement are right-heart-sided endocarditis, tumours, or masses. Relative contraindications for PAC placement include severe coagulopathy including severe thrombocytopenia, presence of a tricuspid or pulmonary valve prosthesis, new pacing lead, and large atrial septal defect. PAC insertion in patients with a left bundle branch block may induce complete heart block. In patients with TR, catheter passage might be more difficult [125]. Clearly, contraindications related to central venous cannulation, including skin infections and thrombosis of the selected vein, apply to PAC insertion as well.

## Conclusion

The contemporary PAC provides accurate and continuous measurements of CO, RV performance, and of the balance between DO_2_ and VO_2_. It provides a multi-variable integration of hemodynamic data in daily clinical practice. Thorough understanding of these PAC-derived measurements and its limitations are key to the successful application of the PAC in clinical practice.

## Supplementary Information

Below is the link to the electronic supplementary material.

Supplementary file1 (PNG 616 KB)

## Abbreviations

CO: Cardiac output
CCO: Continuous cardiac output
CCI: Continuous cardiac index
CVP: Central venous pressure
EDV: End-diastolic volume
EDVi: End-diastolic volume index
Hb: Hemoglobin
HFpEF: Heart failure with preserved ejection fraction
ICU: Intensive care unit
LV: Left ventricle
LVEDP: Left ventricular end-diastolic pressure
LVEDV: Left ventricular end-diastolic volume
MAP: Mean arterial pressure
MPAP: Mean pulmonary artery pressure
MRI: Magnetic resonance imaging
PA: Pulmonary artery
PAC: Pulmonary artery catheter
PAP: Pulmonary artery pressure
PAPi: Pulmonary artery pulsatility index
PAWP: Pulmonary artery wedge pressure
PEEP: Positive end-expiratory pressure
PH: Pulmonary hypertension
RV: Right ventricle
RVEDV: Right ventricular end-diastolic volume
RVEF: Right ventricular ejection fraction
ScvO_2_: Central venous oxygen saturation
SvO_2_: Mixed venous oxygen saturation
SV: Stroke volume
SVi: Stroke volume index
TR: Tricuspid regurgitation
VO_2_: Systemic oxygen consumption
